# Adenosine Signaling in Primary and Metastatic Brain Tumors: Immune Suppression, Tumor Progression, and Therapeutic Opportunities

**DOI:** 10.1007/s12035-026-05930-9

**Published:** 2026-05-21

**Authors:** Tiago de Oliveira Lima, Adinei Abadio Soares, Bernardo Ribeiro Böhm, Bruna Marciano Teixeira, Luiz Cláudio Teixeira da Costa, Kailane Paula Pretto, João Victor Garcia de Souza, Marcelo Lemos Vieira da Cunha, Claudiana Lameu, Detlev Boison, Débora Tavares de Resende eSilva

**Affiliations:** 1 Department of Fundamental Chemistry, University of São Paulo, São Paulo, Brazil; 2 Department of Environmental Engineering, Federal University of Rondônia, Ji-Paraná Campus, Ji-Paraná, Rondônia Brazil; 3 Department of Medicine, Federal University of Fronteira Sul (UFFS), Chapecó, Santa Catarina Brazil; 4 Department of Medicine, Federal University of Uberlândia – UFU, Uberlândia, Brazil; 5 Department of Medicine, Federal University of Catalão – UFCAT, Catalão, Brazil; 6 Department of Nursing, Federal University of Fronteira Sul (UFFS), Chapecó, Santa Catarina Brazil; 7 Department of Graduate Studies in Biomedical Sciences, Federal University of Fronteira Sul (UFFS), Chapecó, Santa Catarina Brazil; 8 IEP - Hospital Sírio Libanês, São Paulo, Brazil; 9 Hospital das Clínicas of the Federal University of Paraná, Curitiba, Brazil; 10 Santa Catarina Society of Neurosurgery, Chapecó, Santa Catarina, Brazil; 11 Biochemistry Department, Institute of Chemistry, University of São Paulo, São Paulo, Brazil; 12 Department of Neurosurgery, Robert Wood Johnson Medical School, Rutgers University, Piscataway, NJ USA; 13 Brain Health Institute, Rutgers University, Piscataway, NJ USA; 14 Rutgers Cancer Institute, Rutgers University, New Brunswick, NJ USA

**Keywords:** Adenosine, Cancer, Purinergic signaling

## Abstract

Brain tumors, whether primary or metastatic, remain a significant cause of cancer-related mortality and are characterized by a profoundly immunosuppressive tumor microenvironment (TME). Among the signaling pathways that shape this environment, purinergic signaling—particularly the adenosine (ADO) axis—has emerged as a central regulator of tumor progression. In the TME, extracellular ATP (eATP) is sequentially converted into ADO by the ectonucleotidases CD39 and CD73, leading to sustained activation of adenosine P1 receptors. This review examines the receptor-specific roles of A1, A2A, A2B, and A3 receptors in modulating neuroinflammation, immune evasion, and therapeutic resistance across distinct brain tumor entities. At low extracellular ADO concentrations, high-affinity A1 and A2A receptors dominate signaling; A1 receptor (A1R) activation predominantly exerts neuroprotective effects by limiting glutamatergic excitotoxicity and preserving blood–brain barrier (BBB) integrity. However, under pathological conditions characterized by sustained ADO accumulation, this homeostatic balance is disrupted. Elevated ADO levels progressively engage low-affinity A2B and A3 receptors, while prolonged A2A receptor activation shifts from regulatory to immunosuppressive signaling, collectively driving T-cell dysfunction, angiogenesis, glioblastoma stem-like cell maintenance, and metastatic competence. Taken together, these findings highlight that pathological ADO receptor engagement in brain tumors is governed by extracellular ADO availability and receptor affinity, supporting a pharmacological framework in which selective inhibition of A2B and A3 receptors, combined with context-dependent modulation of A2A and preservation of A1R-mediated neuroprotective signaling, represents a rational strategy to limit tumor progression while minimizing adverse neurological effects.

**Supplementary Information:**

The online version contains supplementary material available at 10.1007/s12035-026-05930-9.

## Introduction

Millions of new cancer cases are predicted annually worldwide [1–3]. These malignant tumors remain among the leading causes of disease-related deaths [3], with brain tumors associated with particularly high mortality rates. As brain tumors can be either primary or secondary, their origins vary, and consequently, the pathophysiology is specific to each tumor type. Notwithstanding their divergent characteristics, the evidence suggests that certain properties are likely to be shared among these tumor types, given the specialized brain microenvironment. Moreover, the effective targeting of brain tumors is often challenging, and novel strategies to address brain tumor-associated mechanisms involve modulation of signaling within the TME. It is well established that cancerous cells hijack a wide range of physiological processes in the TME, exploiting signals from immune and stromal cells to support their metabolism, with purinergic signaling playing a key role in brain tumor progression.

Purinergic signaling is mediated through receptors, which are classified into the P1 receptors, A1, A2A, A2B, and A3 activated by adenosine (ADO), and P2 receptors, activated by eATP and other nucleotides such as ADP and UTP, P2X1-P2X7, and P2Y [1, 2, 4, 6, 11–14]. These receptors are located on the cell membrane and perform essential functions in the healthy central nervous system (CNS), but they can also modulate pro-tumor processes. In the context of chronic inflammation within the TME, the tissue becomes enriched in eATP, which is sequentially hydrolyzed into ADP, AMP, and ADO by the activity of ectonucleoside triphosphate diphosphohydrolase-1 (CD39) and 5′-ectonucleotidase (CD73). Thus, extracellular ADO levels are mainly regulated by CD39 and CD73, whose overexpression is often associated with an immunosuppressed microenvironment [4].

In the healthy brain, microglia stands out as the cell with the most significant influence on the hydrolysis of ATP into ADO [5]. On the other hand, in the TME, the expression of ectonucleotidases is often dysregulated, and several cells overexpress these components, contributing to ADO-mediated events [6–8]. Thus, purinergic signaling, particularly the ATP-to-ADO degradation pathway, has emerged as a relevant mechanism in brain tumor biology. The mechanisms through which ADO accumulation shapes immune evasion and therapeutic resistance in the brain microenvironment has been reviewed only in specific contexts of glioma, such as glioblastoma [9–11]. However, it remains unclear whether these mechanisms are conserved among different brain tumors or whether ADO signaling may depend on tumor type, e.g., gliomas or breast cancer–derived metastases. Therefore, this review aims to examine the role of ADO in primary and metastatic brain tumors, with a particular focus on its contribution to inflammation and tumor progression. In addition, we propose new interpretations of receptor-specific and tumor-dependent mechanisms through which ADO signaling may drive the brain TME toward immunosuppression, and discuss how these processes can inform the development of novel therapeutic strategies in cancer treatment.

## Adenosine and Tumor-Associated Neuroinflammation

Within the immunosuppressed TME, ADO signaling emerges as a central regulatory axis, promoting tumor-host interactions [12]. Rather than acting as an isolated inflammatory mediator, ADO functions as a context-dependent modulator of immune, metabolic, and vascular processes within the brain. Under physiological conditions, transient inflammatory responses support neuronal protection and tissue repair, with microglia and macrophages contributing to synaptic regulation and immune surveillance. In brain tumors, however, these regulatory mechanisms are progressively distorted by sustained alterations in purinergic signaling [13].

Extracellular ADO levels are tightly regulated by intracellular adenosine kinase (ADK), which phosphorylates ADO to AMP, and transmembrane transport, thereby contributing to the equilibrium between intra- and extracellular ADO pools. In many tumors, ADK expression and ADO metabolism are dysregulated, contributing to abnormal accumulation of extracellular ADO and persistent activation of P1 receptors [14–16]. This pathological receptor engagement does not simply amplify inflammatory signaling but instead drives a shift toward immune suppression, altered cellular communication, and tumor-permissive signaling networks. Chronic low-grade inflammatory signaling should be viewed as a permissive feature of the brain TME rather than as an independent pathological endpoint. Sustained ADO accumulation influences tumor progression, therapy resistance, and immune escape by selectively engaging adenosine receptor subtypes with distinct affinities and signaling properties. Accordingly, the following sections focus on how individual P1 receptor subtypes contribute to tumor progression and immune regulation in primary and metastatic brain tumors, rather than addressing neuroinflammation as a standalone process.

### Adenosine Receptors

**Fig. 1:**
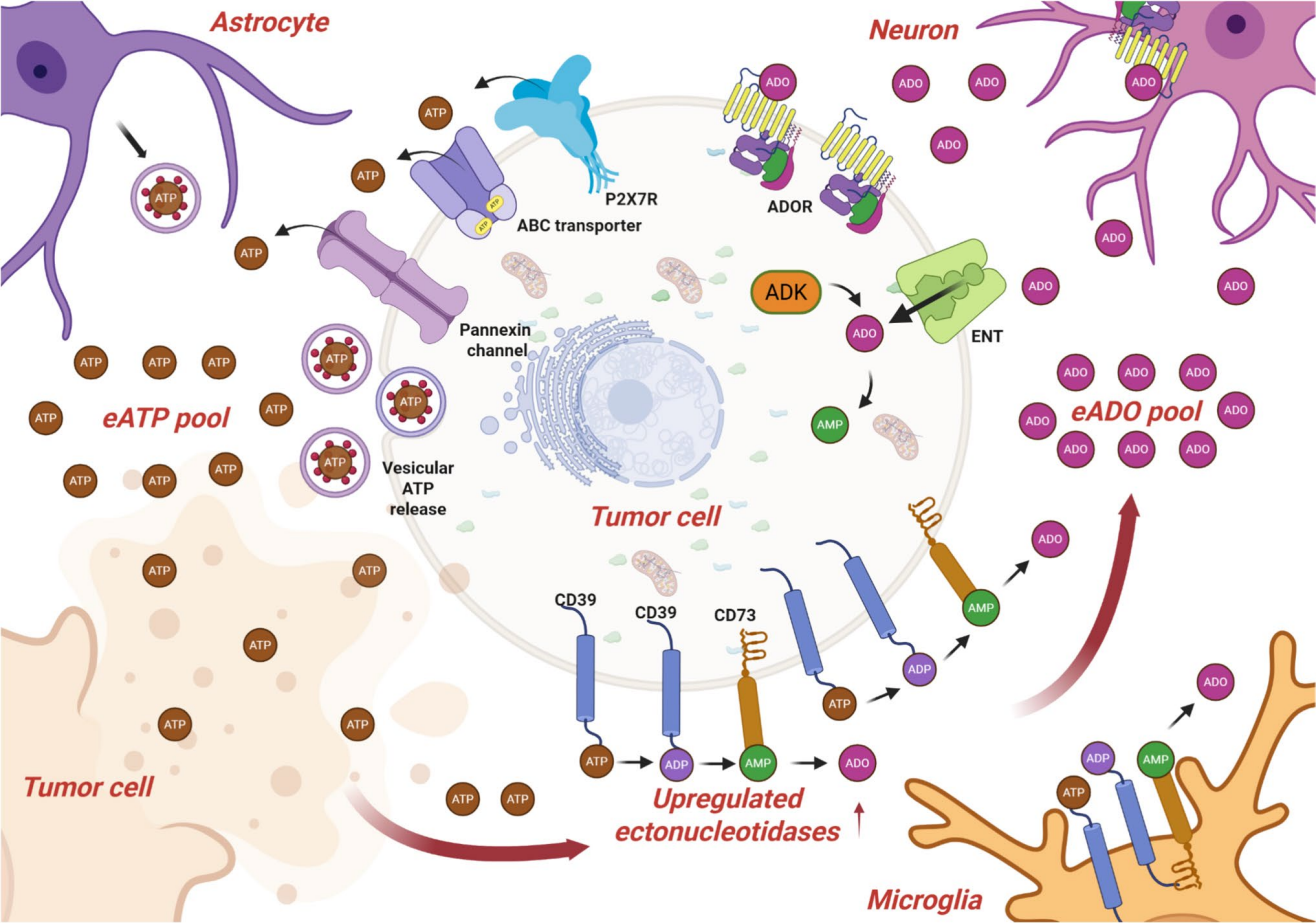
ATP and adenosine dynamics in the tumor microenvironment. Extracellular ATP (eATP) accumulates in the tumor microenvironment through multiple mechanisms, including vesicular release, pannexin channel-mediated efflux, and transport via ATP-binding cassette (ABC) transporters. Tumor cells, astrocytes, and other surrounding cells contribute to the eATP pool, which can also be amplified by P2X7 receptor activation, promoting further ATP release. Once in the extracellular space, eATP is sequentially hydrolyzed by upregulated ectonucleotidases, primarily CD39 and CD73, generating ADP, AMP, and extracellular adenosine (eADO). The resulting eADO pool is shaped by both enzymatic activity and transport processes, including equilibrative nucleoside transporters (ENT) and intracellular metabolism via adenosine kinase (ADK). Adenosine then signals through adenosine receptors (ADORs) expressed across multiple cell types, including neurons, microglia, astrocytes, and tumor cells, contributing to the modulation of tumor growth, immune responses, and neuro-glial interactions within the brain tumor microenvironment. Created with BioRender.com

ADO levels are highly dependent on extracellular ATP (eATP) concentrations within the TME. In the brain, multiple mechanisms contribute to the release of eATP (Fig. 1). These include (i) vesicular release, in which ATP is released as a danger-associated signal to communicate cellular stress to neighboring cells [17]; (ii) membrane disruption associated with cell death, particularly under therapeutic stress, which promotes ATP leakage into the extracellular space [18]; (iii) ATP-binding cassette (ABC) transporters, which may contribute to eATP accumulation and are often upregulated in drug-resistant cells [19], a feature commonly observed in brain tumors; (iv) pannexin channels, which mediate the release of small signaling molecules, including ATP, into the extracellular space [20]; and (v) P2X7 receptor activation, which has been associated with pore formation and may contribute to increased extracellular ATP levels in brain tumors [21].

Once released, eATP is sequentially converted into ADO by the ectonucleotidases CD39 and CD73, whose expression is frequently upregulated in brain tumors and contributes to the establishment of an immunosuppressive microenvironment [22, 23]. Notably, ectonucleotidases expressed under physiological conditions, particularly in astrocytes and microglia, may also contribute to ADO pool enrichment within the tumor microenvironment, potentially supporting tumor-promoting processes [24]. In parallel, ADO levels are further regulated by equilibrative nucleoside transporters (ENTs) and ADK, which control its extracellular availability [25]. In this context, the coordinated activity of these pathways promotes ADO accumulation and sustained P1 receptor stimulation.

Each P1 receptor subtype exhibits a distinct affinity for ADO, with A1 and A2A classified as high-affinity receptors and A2B and A3 as low-affinity receptors [26]. As ADO levels increase several-fold in the TME, all P1 receptor subtypes may contribute to tumor progression in distinct ways [27]. All P1 are G protein–coupled receptors. A1 and A3 act as inhibitory receptors through Gi/Go proteins, whereas A2A functions as excitatory receptors via Gs. A2B, in turn, is often described in the literature as a pleiotropic receptor, as it can couple to multiple G proteins, primarily Gs and Gq, and in some contexts Gi, leading to context-dependent signaling outcomes [28, 29]. A1 and A3 receptors inhibit the production of cyclic adenosine monophosphate (cAMP) by adenylate cyclase (AC), in contrast to A2A and A2B, which increase cAMP levels. These mechanisms are particularly relevant in the context of cancer progression, as cAMP levels can shape the activation of different signaling pathways that contribute to processes such as immune evasion, cell proliferation, and immunosuppression.

Given that malignancies encompass a wide spectrum of heterogeneous diseases arising in distinct biological and clinical contexts, it is not expected that P1 receptors behave uniformly across all tumor types. Nevertheless, the idea that these receptors are frequently associated with tumor progression is well supported by the literature [12, 30–32]. The following section highlights the main evidence indicating that P1 receptors play a key role in the progression of brain tumors, especially glioblastoma and breast cancer brain metastasis.

## A1 Receptor

The A1 receptor is predominantly expressed in the CNS, but it is also present in other organs such as adipose tissue, liver and in the cardiac atria [33], (https://pmc.ncbi.nlm.nih.gov/articles/PMC6327119/). A1R signals through the Gi/o system, and its activation promotes Gβγ-mediated activation of GIRK channels [34]. This leads to potassium efflux and membrane hyperpolarization, which reduces the opening of voltage-dependent Ca [2]⁺ channels, thereby decreasing glutamate release. By limiting excessive activation of N-methyl-D-aspartate (NMDA) receptors, this mechanism helps prevent excitotoxicity [35]. Through this pathway, A1R exerts a well-established neuroprotective role by controlling excitatory neurotransmission and calcium-dependent neuronal stress. Although Gs- and Gq-coupled receptors also dissociate into Gα and Gβγ subunits, the activation of Gβγ-dependent ion channels appears to be more prominent in Gi/o signaling, likely due to differences in kinetic and spatiotemporal dynamics at the cell membrane [36].

However, glioma and brain-metastatic cells are known to benefit from glutamatergic signaling, which can be co-opted to support tumor cell proliferation and migration through NMDA receptor activation, as well as through AMPA receptor–mediated synaptic integration into neural circuits [37, 38]. Moreover, glutamate accumulation may modulate microglial plasticity and, in association with growth factors within the brain TME, promote phenotypic states associated with tumor-supportive functions, including extracellular matrix remodeling, maintenance of hypoxic conditions, and the release of eATP [39].

Thus, although A1R acts as a homeostatic regulator of glutamatergic signaling under physiological conditions, it may also influence excitatory dynamics in the pathological context of brain tumors. Accordingly, while A1R signaling impacts immune-related processes in the brain TME, it should not be interpreted as a classical immune checkpoint, but rather as an upstream modulator of neuronal excitability and purinergic-driven neuroinflammatory signaling.

The increase in eATP promoted by tumor cells also results in the accumulation of ADO, leading to greater expression of P1 receptors in the high-grade cancer setting [40, 41]. That is, in peritumoral parenchymal tissue and glioblastoma cells, there is increased protein expression of A1R compared to homeostatic tissue [42]. The neuroprotective effects of A1R are well-established in cases of injury or non-sustained toxicity, but are not yet fully understood in chronic settings, such as those seen in CNS neoplasms [26].

Cunha (2016) addresses the impact of using A1R agonists for neuroprotection and attempts to reduce glutamate release, and postulates that A1R undergoes a loss of action resulting from sustained increases in ADO levels in the brain parenchyma due to lesions in neurodegenerative models. After the maintenance of noxious stimuli and subsequent elevation of ADO, the administration of an A1R agonist agent was unable to slow down brain damage because the receptor underwent desensitization [43]. The maintenance of the neuroprotective effect of A1R, in order to limit glutamate release in the context of TME in established cancer, is not guaranteed at the time of writing this article, and further investigation is needed to determine if there would be benefits from the development of A1R agonists [26, 44].

Furthermore, A1R-mediated attenuation of NMDA receptor activity, due to the directly proportional relationship between glutamate and NMDA, is particularly relevant, as NMDA signaling has been shown to be functionally active in metastatic breast-to-brain cancer cells [45]. Excessive NMDA activation is associated with increased blood–brain barrier (BBB) permeability and facilitation of metastatic cell extravasation in multiple experimental models [46], whereas pharmacological NMDA antagonism decreases BBB permeability [47], indicating that both hyperactivation and full blockade of NMDA receptors can disrupt BBB homeostasis. In this biochemical context, A1R signaling may function upstream by modulating presynaptic glutamate release and excitotoxic tone, thereby contributing to the dynamic regulation of NMDA receptor activity and BBB permeability within the brain TME.

Moreover, glutamatergic and calcium-dependent signaling pathways are well-established drivers of glioma initiation [48]. Sustained excitatory signaling supports metabolic reprogramming, proliferation, and invasive behavior of tumor cells. As many brain tumors exploit glutamatergic stimulation as a mechanistic driver [39, 49, 50], A1R activation emerges as a potentially advantageous strategy: by inhibiting glutamate release upstream, A1R activation may interfere with metabolic and calcium-dependent pathways required for tumor progression while avoiding the broader disturbances in physiological neuronal function associated with direct NMDA receptor inhibition. In line with this rationale, targeted activation of A1R signaling within tumor tissue represents a mechanistically grounded strategy that merits further investigation.

Regarding immune modulation through A1R activation, it is known that A1R co-localizes with the regulator of G protein signaling 4 (RGS4), which downregulates A1R through neurabin-mediated action in the brain. In this context, Wang’s group identified a peptide that disrupts the neurabin interaction within the A1R–RGS4 complex, thereby enabling A1R stimulation. This approach is relevant because it prevents peripheral A1R activation outside the CNS while enhancing the protective effects of A1R stimulation in the context of brain tumors [51, 52].

Moreover, Daniele and colleagues showed that A1R activation may provide therapeutic benefit in the treatment of glioblastoma when combined with temozolomide (TMZ) [53]. The group demonstrated that A1R potentially acts by sensitizing glioblastoma cancer stem cells (GSCs) to treatment and by activating pro-apoptotic pathways in tumor cells, thereby attenuating their proliferation. This mechanism positions A1R as promising in new strategies to overcome resistance to chemotherapy, which is one of the major challenges in the treatment of brain tumors.

A1R signaling has been also associated with cell-cycle regulation and proliferation in pancreatic cancer [54]. However, evidence specifically addressing this role in brain tumors remains limited. For instance, Bi and colleagues reported that treatment with Paris saponin H (PSH) induced cell-cycle arrest in U251 glioblastoma cells in an A1R-dependent manner [55]. While these findings suggest a potential link between A1R and brain tumor cells, further studies are required to clarify its role in tumor proliferation within the brain TME.

On the one hand, metastatic cells, such as those from breast cancer, can migrate to and colonize the brain due to factors that favor tumor growth, including the establishment of an immunosuppressive microenvironment driven by the pro-tumoral activity of M2 microglia, which promotes immune escape [56]. On the other hand, the A1R receptor expressed in microglia may orchestrate mechanisms associated with disease, inflammation, and changes in the central nervous system microenvironment [57]. This is particularly relevant because excessive neuroinflammation creates a microenvironment that supports the growth and progression of brain metastases [58]. Moreover, uncontrolled inflammation in surgical or radiotherapy contexts has been reported as a detrimental factor affecting treatment efficacy, prognosis, and therapeutic control of brain metastases [59, 60]. In the context of inflammatory regulation, A1R can modulate activated microglia (triggered by factors such as TNF-α, IL-1β, and IFN-γ) to control and reduce the neuroinflammatory response. This suppression of microglial activity helps limit the excessive and damaging effects of inflammation in central nervous system lesions [61]. Thus, stimulation of A1R in the neural microenvironment plays an important protective role in the central nervous system [61, 62].

Furthermore, the safety of potential therapeutic applications involving P1 receptor agonists and antagonists must be carefully considered, as adenosine receptor antagonists are generally regarded as safer than available agonists due to the latter’s extensive adverse effects [63]. Nevertheless, studies to date show that A1R agonists are effective against neurodegeneration in a prophylactic and punctual manner, but without testing in cancer settings [43]. Importantly, ADO signaling in the brain TME is mediated by multiple receptor subtypes, whose distinct signaling properties may differentially shape neuronal, inflammatory, and tumor-associated processes [64].

## A2 Receptor

Historically, adenosine A2 receptors (A2Rs) were distinguished from A1R based on their pharmacological effects on adenylate cyclase (AC), with A2R activation stimulating AC and A1R activation inhibiting it. Because the signaling output of A2A and A2B receptors initially appeared similar, both being considered activators of AC, their distinction relied primarily on pharmacological criteria, particularly their affinity for ADO, with A2AR classified as a high-affinity receptor and A2BR as a low-affinity receptor. However, receptor cloning studies later demonstrated that A2B receptors can couple to a wide range of G proteins, including Gs, Gq, and less frequently Gi, whereas A2A receptors are consistently coupled to Gs [65]. Thus, A2AR exhibits functional predictability, producing increases in cAMP via stable Gs coupling, whereas A2BR displays signaling plasticity, engaging multiple G proteins and rendering cAMP accumulation conditional on receptor density, cellular context, and microenvironmental cues.

cAMP signaling plays a crucial role in tissue homeostasis, primarily by activating protein kinase A (PKA) but also by engaging other effectors such as exchange proteins directly activated by cAMP (EPAC), thereby contributing to cellular processes including proliferation, metabolism, gene expression, and cell death [66]. Tumor cells, however, hijack physiological signaling to enhance survival, including cAMP-related pathways. Because Gs-coupled receptors activate AC to increase intracellular cAMP, one might expect A2A and A2B receptors to produce similar responses. Nevertheless, because cAMP function depends on the spatial and temporal organization of the cytoplasm [67, 68], each receptor may engage distinct signaling components and ultimately trigger different cellular outcomes [69]. On this basis, the following sections discuss the distinct roles of A2AR and A2BR in brain tumors, highlighting how their differential signaling properties shape neuroinflammatory and tumor-associated processes.

## A2A Receptor

In the healthy brain, A2ARs are particularly abundant in striatal GABAergic neurons, where they regulate neurotransmission, synaptic plasticity, and basal ganglia–dependent behaviors [70]. Under specific physiological conditions, A2AR activity can oppose A1R effects. For example, A2AR activation enhances neurotransmitter release in the context of recent lesions, particularly within the first two weeks after injury [71]. Although both are considered high-affinity adenosine receptors, the A2AR exhibits lower sensitivity to ADO than A1R, and their activation therefore triggers distinct cellular responses [72]. This difference supports the idea that low extracellular ADO concentrations maintain homeostatic and neuroprotective pathways (primarily via the A1R), whereas elevated ADO levels, typical of tissue damage or chronic inflammation, preferentially engage A2ARs and thereby signal cellular distress.

Mechanistically, as described earlier, A2AR signals through Gs protein activation. Upon binding of extracellular ADO, A2AR undergoes a conformational change that promotes the exchange of GDP for GTP on the Gαs subunit, leading to activation of AC. Activated Gαs stimulates AC, resulting in the conversion of ATP to cAMP, which in turn activates PKA. PKA phosphorylates serine and threonine residues within specific consensus sequences in a wide range of downstream targets [73].

Additionally, emerging evidence indicates that Gs-coupled GPCRs can also modulate intracellular Ca^2^⁺ signaling through Gβγ-mediated activation of phospholipase Cβ (PLCβ), leading to cytosolic Ca^2^⁺ release [74]. This mechanism, which may operate in parallel with cAMP-dependent pathways, highlights the potential cross-talk between A2AR-mediated Gs signaling and calcium dynamics. Given the established role of Ca^2^⁺ signaling in processes such as angiogenesis and tumor progression [75], this cross-talk may further contribute to the pro-tumorigenic effects of adenosine signaling in the tumor microenvironment.

A2AR has been reported to increase BBB permeability and promote lymphocyte influx into the CNS [76, 77]. Although increased lymphocyte trafficking into the CNS is often interpreted as a sign of effective antitumor immunity, in an ADO-rich brain TME this infiltration becomes functionally misleading, as A2AR–mediated signaling potently suppresses T-cell activation, cytotoxicity, and cytokine production, thereby converting immune infiltration into an immunosuppressive, tumor-permissive state [78]. Accordingly, tumors exploit A2AR engagement, taking advantage of the ADO-rich environment to promote immunosuppression and BBB dysfunction. In this context, circulating tumor cells (CTCs) may exploit A2AR-mediated increases in vascular permeability to enhance BBB transmigration and establish crosstalk with the brain stroma. Together, these mechanisms suggest that A2AR activation is tightly associated with brain tumor progression and may facilitate metastatic spread to the brain.

Outside the brain, A2ARs are highly expressed in multiple immune cell populations, particularly T lymphocytes where A2AR signaling constitutes a major regulatory pathway of lymphocyte activation. In line with its role as a sensor of elevated extracellular ADO, A2AR activation predominantly exerts immunosuppressive effects, reducing T-cell effector function and antitumor immune surveillance [79, 80]. In the TME, this peripheral immunomodulatory profile limits the capacity of lymphocytes to recognize tumor cells and restrain their dissemination during the metastatic cascade, providing the rationale for targeting A2AR signaling as an immunotherapeutic strategy [81].

In addition to its effects on lymphocytes, A2AR induces a metabolic shift in peripheral macrophages via IL-10 production [82], thereby promoting polarization toward an M2-like phenotype and favoring a pro-tumorigenic immune environment [83]. However, despite these observations, the role of A2AR signaling within CNS immune cell networks remains poorly understood in brain tumors, representing an important area for further investigation. Notably, these observations may offer valuable insights to guide future studies in the CNS context, particularly given the functional parallels between peripheral and brain-resident immune cells. Moreover, levels of A2AR expression across distinct immune cell subpopulations within the CNS remain insufficiently characterized.

By contrast, in vivo studies supported a tumor-suppressive role of A2AR during early stages of brain metastasis establishment through SDF-1/CXCR4 inhibition [84, 85]. The SDF-1 (CXCL12)/CXCR4 chemokine axis is a well-established driver of tumor cell chemotaxis, invasion, and metastatic homing to distant organs [86].

Furthermore, in vitro studies and those replicated in mice by Lei Chen et al. (2020) have demonstrated that A2AR stimulation represents a promising therapeutic target to be investigated in the control of brain metastases, due to its potential to suppress the SDF-1/CXCR4 axis, which is known for its strong ability to promote and direct metastatic cells [85]. This occurs because activated A2AR downregulates the expression of SDF-1 and CXCR4, thereby reducing tumor chemotactic capacity, inhibiting cancer cell proliferation and viability, and contributing to the maintenance of BBB integrity, ultimately preventing brain metastasis. In addition, the study demonstrated that activated A2AR are capable of stabilizing the BBB, enhancing its protective role in isolating the central nervous system from metastatic invasion.

In contrast, a Phase I/II clinical study in humans by Jackson et al. (2023) investigated whether activation of the A2AR, through the agonist regadenoson, could modulate the BBB to facilitate the delivery of glioblastoma therapies [87]. In this study, A2AR activation did not significantly modulate the BBB. However, the study included a small sample size (six patients with glioblastoma), and the authors noted that higher doses than 0.4 mg of regadenoson might yield different results. These factors may have influenced the findings, highlighting the need for further studies to better elucidate the role of A2AR in regulating the BBB. Finally, it is important to note that A2AR has been widely investigated for its antitumor potential, both in preventing metastasis and in regulating the BBB during cancer treatment. Nevertheless, dysregulated and excessive A2AR activity has also been described in the literature as an important pro-tumoral factor.

These distinct roles of A2AR in tumor biology are probably linked to tumor heterogeneity and suggest that A2AR-targeted therapies may need to follow a personalized, CAR-T–like approach rather than a generic treatment strategy. Taken together, these observations highlight the inherent dual challenge of targeting A2AR signaling, which operates through distinct mechanisms in the brain and in peripheral immunity in not-yet-fully-understood ways.

## A2B Receptor

Unlike the A2AR, the A2B receptor (A2BR) is expressed at a significantly lower density in brain regions. Consequently, it has been historically considered to be predominantly a pathophysiological receptor, given the low ADO levels observed in healthy tissues. However, A2BR sensitivity increases under conditions of overexpression, indicating that the receptor may become responsive even at nanomolar concentrations [88]. Moreover, beyond the CNS, the A2BR displays broad tissue distribution, including the intestine, vasculature, and, most prominently, the immune system, where it is highly enriched in macrophages [89].

Mechanistically, A2BR activation engages multiple downstream pathways, consistent with its well-established pleiotropic signaling profile. As Gi- and Gs-coupled signaling have already been discussed for A1R and A2AR, respectively, we now turn our attention to Gq signaling, which stimulates phospholipase C (PLC-β). This mechanism triggers an IP₃-dependent increase in intracellular calcium levels and may subsequently activate protein kinase C (PKC) [90]. Calcium flux, in turn, acts as an important second messenger and regulates processes such as cytoskeletal remodeling, cell migration and invasion, resistance to apoptosis, and sustained proliferative signaling [91, 93–96]. Thus, prolonged A2BR activation in brain cells may contribute to the development and progression of brain tumors.

Consistent with these mechanisms, in the context of the brain TME, A2BR has been shown to stimulate IL-6 expression in murine microglia via PLC activation under hypoxic conditions [97], further supporting the role of A2BR-mediated signaling in linking calcium-dependent pathways to pro-inflammatory and pro-tumorigenic responses.

IL-6, in turn, has been implicated as a mediator of interactions between brain-tropic tumor cells and M2-like microglia during the establishment of brain metastases in non-small cell lung cancer (NSCLC) [98]. Thus, A2BR activation in microglia may represent a mechanism by which ADO contributes to the orchestration of a tumor-supportive microenvironment, promoting sustained signaling and adaptation of metastatic cells within the brain. However, further studies are required to clarify whether IL-6 production by tumor-associated microglia is directly regulated by A2BR signaling. In addition, A2BR expression in antigen-presenting cells has been associated with CD8^+^ T cell exhaustion and tumor growth [99], highlighting its contribution to tumor maintenance and progression across different cell types.

Bynoe’s team investigated the role of A2BRs in glioblastoma progression and demonstrated that CD73 contributes to tumor aggressiveness by generating high local concentration of ADO to engage the low-affinity A2BR, thereby triggering metalloproteinase-2 (MMP-2)-dependent signaling cascades. Moreover, A2BR inhibition enhances the responsiveness of glioblastoma cells to therapeutic drugs, helping to overcome treatment resistance [100]. However, it is important to note that the modulation of MMP-2 is not exclusive to A2BR. Although it has been demonstrated that A2BR can stimulate MMP-2 signaling, other studies have also indicated that, in the tumor or metastatic context, the modulation of the MMP-2 pathway may be influenced by several other components of purinergic signaling, such as the activation of P2 family receptors, CD73 levels, or ADO concentrations in the microenvironment [101, 102].

Consistent with its pleiotropic signaling profile, A2BR shares overlapping downstream pathways with other P1 receptors, particularly those related to Gs-mediated signaling. Specifically, A2BR cooperates with A2AR to sensitize the interaction between GGTase-I and Rap1B through PKA activation [103]. This mechanism is believed to be targeted by specific miRNAs to sustain cancer cell survival and migration, thereby promoting tumor aggressiveness and contributing to metastatic spread [103, 104]. Collectively, the evidence suggests that A2BR antagonism represents a potent strategy to support cancer therapy. As a final point, although A2BR does participate in normal physiological processes, its functions appear to be less functionally ambiguous than those of A2AR. Thus, A2BR emerges as a strong and comparatively straightforward therapeutic target for brain tumors and potentially several other malignancies.

## A3 Receptor

In humans, the A3 receptor (A3R) is highly expressed in immune cells, cardiac tissue, epithelial cells, the colonic mucosa, lung parenchyma, and the bronchial epithelium [105]. In immune cells, A3R modulation regulates cytokine production,, chemotaxis, cytotoxicity, apoptosis, and proliferation [106], supporting its involvement in inflammatory processes, particularly in chronic inflammatory pathologies, ranging from autoimmune diseases to the orchestration of the TME [107].

Within TME, hypoxia can be exploited by tumor cells, though intrinsically cytotoxic, it also drives adaptive programs that support tumor progression. Evidence indicates that A3R activation contributes to angiogenesis by inducing VEGF transcription, thereby promoting neovascularization [108]. In parallel, activation of A3R has been shown to induce the release of early growth factors [109], suggesting a role of this receptor in the establishment of pro-adaptative microenvironment. By modulating vascular remodeling, A3R influences nutrient and oxygen availability which may facilitate tumor-stroma interactions associated with disease progression [108].

Mechanistically, A3R activation has been shown to engage canonical oncogenic signaling pathways, including MEK/ERK1/2 and PI3K/Akt [106, 110]. In glioblastoma models, these pathways converge on functional outputs related to cell survival and drug resistance, including the regulation of the multidrug resistance-associated protein 1 (MRP1) transporter. In addition, A3R signaling has been associated with activation of p38 MAPK and c-Jun N-terminal kinase (JNK), promoting increased cell migration and upregulation of matrix metallopeptidase-9 (MMP-9), a key mediator of extracellular matrix remodeling and invasion [106, 110]. Notably, pharmacological antagonism of A3R has the potential to reduce tumor volume [106, 108].

These signaling effects are especially relevant within hypoxic niches enriched in GSCs. These hypoxic regions are characterized by elevated extracellular ADO levels driven by the activity of the CD73 and ACP3 (PAP), and the activation of A3R [108, 111, 112].

In the CNS, these mechanisms are further refined by the neural microenvironment. Brain tumor progression is shaped by neuronal activity, glutamatergic signaling, and neurovascular dynamics, which modulate pathways such as MAPK/ERK, JNK, and VEGF beyond their roles in general oncogenesis. For instance, neuronal activity-dependent calcium signaling can activate MAPK/ERK and JNK cascades [113], while hypoxia-driven VEGF signaling contributes to angiogenesis and BBB disruption [108]. In parallel, brain tumors exploit specialized niches, including perivascular regions, where interactions with endothelial cells and neural progenitor-like programs sustain tumor growth and self-renewal [114]. Although direct evidence for a role of A3R in breast cancer brain metastasis remains limited, its expression in breast cancer cells and its ability to modulate signaling pathways associated with migration, survival, and stress adaptation [106, 108, 110] suggest a potential context-dependent contribution to tumor cell-microenvironment crosstalk in the brain. At present, however, a direct mechanistic link between A3R activation and metastatic competence in this setting has not been fully established.

## Clinical Strategies and Future Perspectives

Accumulating evidence reviewed here indicates that ADO signaling exerts distinct yet convergent effects in primary and metastatic brain tumors, depending on receptor subtype engagement, local ADO availability, and tissue context. In both tumor settings, sustained accumulation of extracellular ADO within the TME preferentially activates low-affinity P1 receptors, thereby promoting immune suppression, angiogenesis, vascular remodeling, and therapeutic resistance. While primary brain tumors, such as glioblastoma, primarily exploit ADO-rich niches to sustain local immunosuppression and invasive growth, metastatic brain tumors additionally benefit from ADO-driven alterations in BBB permeability and tumor–stroma communication that facilitate metastatic seeding and outgrowth within the brain. These shared ADO-dependent mechanisms have positioned the purinergic pathway as an attractive therapeutic target across distinct brain tumor entities.

A widely used strategy involves the blockade of ADO-producing enzymes, such as CD39 or CD73. This approach has been extensively explored in peripheral cancers, including triple-negative breast cancer. However, reducing extracellular ADO levels in the brain is not without risk, as it may promote seizure generation and profoundly affect neural activity [115, 116]. An emerging target for the metabolic regulation of ADO is the intracellular enzyme ADK. Inhibition of ADK is an effective means to increase extracellular ADO, whereas gene therapy approaches designed to enhance ADO metabolism have been proposed as strategies to reduce extracellular ADO levels [117–119]. Importantly, metabolic regulation of ADO in brain cancer must be approached with caution, as decreasing extracellular ADO may increase the risk of neurological dysfunction [116].

A more direct pharmacological strategy involves modulation of adenosine receptors, particularly through selective antagonism of the A2A receptor. This approach is based on preventing adenosine binding to A2ARs, thereby limiting the pro-tumoral and immunosuppressive signaling sustained by elevated extracellular ADO levels within the TME. Although this strategy has received increasing attention, its application to brain tumors remains under active investigation.

Moreover, beyond the immunological axis, modulation of A1 and A3 receptors may also represent relevant therapeutic strategies in brain tumors. As discussed above, A1R signaling exerts predominantly neuroprotective effects. In this context, maintaining ADO at low extracellular concentrations may be beneficial for preserving physiological neuronal signaling, which is frequently co-opted by tumor cells to sustain aberrant neurotransmitter-dependent pathways. From this perspective, coordinated regulation of the CD39/CD73/A1R axis appears critical to favor A1R-mediated protective signaling while minimizing excessive activation of A2R under high ADO conditions. Conversely, blockade of the A3R may be advantageous for limiting angiogenesis and tumor vascularization, as A3R signaling has been associated with hypoxia-driven pro-tumoral responses. These complementary strategies, enhancing A1R-mediated neuroprotective signaling while inhibiting A3R-driven angiogenic pathways, could be combined to prevent residual ADO-dependent activation of pro-tumoral mechanisms within the brain TME.

As reviewed by Solanki et al. [120], the expanding pharmacological landscape of P1 receptor modulators underscores the feasibility of receptor-specific therapeutic strategies in cancer.

In the context of therapeutic target strategies, it is important to highlight that the most frequent metastatic cancers found in the central nervous system (CNS) originate from the lung, followed by breast cancer as the second most common, and melanoma as the third. Other less common metastatic tumors include renal cell carcinoma and colorectal cancer, respectively, in decreasing order of incidence in the CNS. Finally, the rarest CNS metastases arise from lymphomas, gastrointestinal, gynecological, and genitourinary tumors, as well as other malignant neoplasms [121, 143].

Table 1 shows the data compiled from original clinical studies on tumors with metastatic potential to the CNS. The articles were selected based on a review of clinical studies available on the PubMed platform up to 2026 and the review by Antonioli et al. (2023) [142], with the Antonioli et al. study being used only as a guide for identifying relevant experimental articles in the PubMed database up to 2023.

**Table 1:** Origial clinical studies on tumors with metastatic potential to the CNS

DOI of the article	Clinical trial phase	Type of tumor with potential for metastasis to the CNS	Agent	Mechanism of action
10.1007/s00262-023-03430-6124	Phase I	Colorectal adenocarcinoma Pancreatic ductal adenocarcinoma EGFR-mutated non-small cell lung cancer	Oleclumab	Anti-CD73
10.1038/s41591-024-02808-y125	Phase II	Non-small cell lung cancer	Oleclumab	Anti-CD73
10.1136/jitc-2023-007279126	Phase II	Early-stage luminal B breast cancer	Oleclumab	Anti-CD73
10.1158/1078-0432.CCR-24-0499127	Phase Ib/II	Metastatic pancreatic ductal adenocarcinoma	Oleclumab	Anti-CD73
10.1038/s41467-023-42744-y128	Phase I/II	Carcinoma triplo‑negativo da mama localmente avançado ou metastático (TNBC)	Oleclumab	Anti-CD73
10.1136/jitc-2022-005267129	Phase I	Advanced solid tumors (cancer)	Dalutrafusp alfa	Anti-CD73
10.1136/jitc-2022-005802130	Phase I	Advanced solid tumors (cancer)	Mupadolimab (CPI-006)	Anti-CD73
10.1158/2159-8290.CD-23-0436131	Phase I/II	Non-small cell lung cancer	Oleclumabe	Anti-CD73
10.1016/j.xcrm.2025.101973132	Phase I	Advanced breast cancer	Oleclumabe	Anti-CD73
10.1038/s41591-025-03746-z133	Phase II	Resectable non-small cell lung cancer	Oleclumabe	Anti-CD73
10.1016/j.ygyno.2024.06.017134	Phase II	Recurrent epithelial ovarian cancer	Oleclumabe	Anti-CD73
10.1001/jamanetworkopen.2025.18440135	Phase II	Unresectable stage III non-small cell lung cancer	Oleclumabe	Anti-CD73
10.1038/s41416-024-02796-3136	Phase II	Metastatic colorectal cancer	Oleclumabe	Anti-CD73
10.1016/j.radonc.2025.110836137	Phase II	High-risk ER +/HER2 − breast cancer	Oleclumabe	Anti-CD73
10.1016/j.lungcan.2025.108834138	Phase II	Non-small cell lung cancer (metastatic)	Oleclumabe	Anti-CD73
10.1007/s00262-024-03640-6139	Phase II	Metastatic castration-resistant prostate cancer	Oleclumabe	Anti-CD73
10.1158/1078-0432.CCR-22-0612140	Phase Ia/b	Solid tumors (breast, melanoma, kidney, liver, etc.)	AZD4635	Antagonista do A2AR
10.1158/1078-0432.CCR-21-2742141	Phase I	Advanced/metastatic non-small cell lung cancer	Taminadenant	Antagonista do A2AR
10.1080/17576180.2025.2554564142	Phase Ib/II	Prostate carcinoma Triple-negative breast cancer MMR-deficient/MSI-high tumors	TT‑702/TT‑478	Antagonista do A2BR
10.1634/theoncologist.2012-0211143	Phase I/II	Advanced, refractory or metastatic hepatocellular carcinoma (HCC)	CF102/Namodenoson	Agonista seletivo do A3AR

It is important to emphasize that the BBB is a physical barrier between the CNS and the rest of the body, which can hinder the delivery of therapeutic agents, including those investigated in the context of metastases or primary CNS tumors.

Table 2 presents a study on glioblastoma. Although the Phase I/II clinical trial by Jackson et al. (2023) [87] in humans did not demonstrate the efficacy of intravenous regadenoson (0.4 mg, single dose) in modulating the A2A receptor to increase blood–brain barrier permeability and facilitate the entry of therapeutic agents, the authors reported that regadenoson effectively modulated A2A in animal studies. They also recommended further investigations using different dosing strategies in humans. Therefore, studies focusing on therapeutic targets that enhance drug delivery across the human blood–brain barrier may contribute to improved treatment of both primary and secondary tumors in the central nervous system.

**Table 2:** Study on glioblastoma

DOI of the article	Clinical trial phase	Type of tumor	Agent	Mechanism of action
10.1186/s12987-017-0088-8	Phase I/II	Glioblastoma	Regadenoson	A2A agonist to facilitate the entry of temozolomide (a chemotherapeutic agent) into the CNS

## Conclusion

**Fig. 2:**
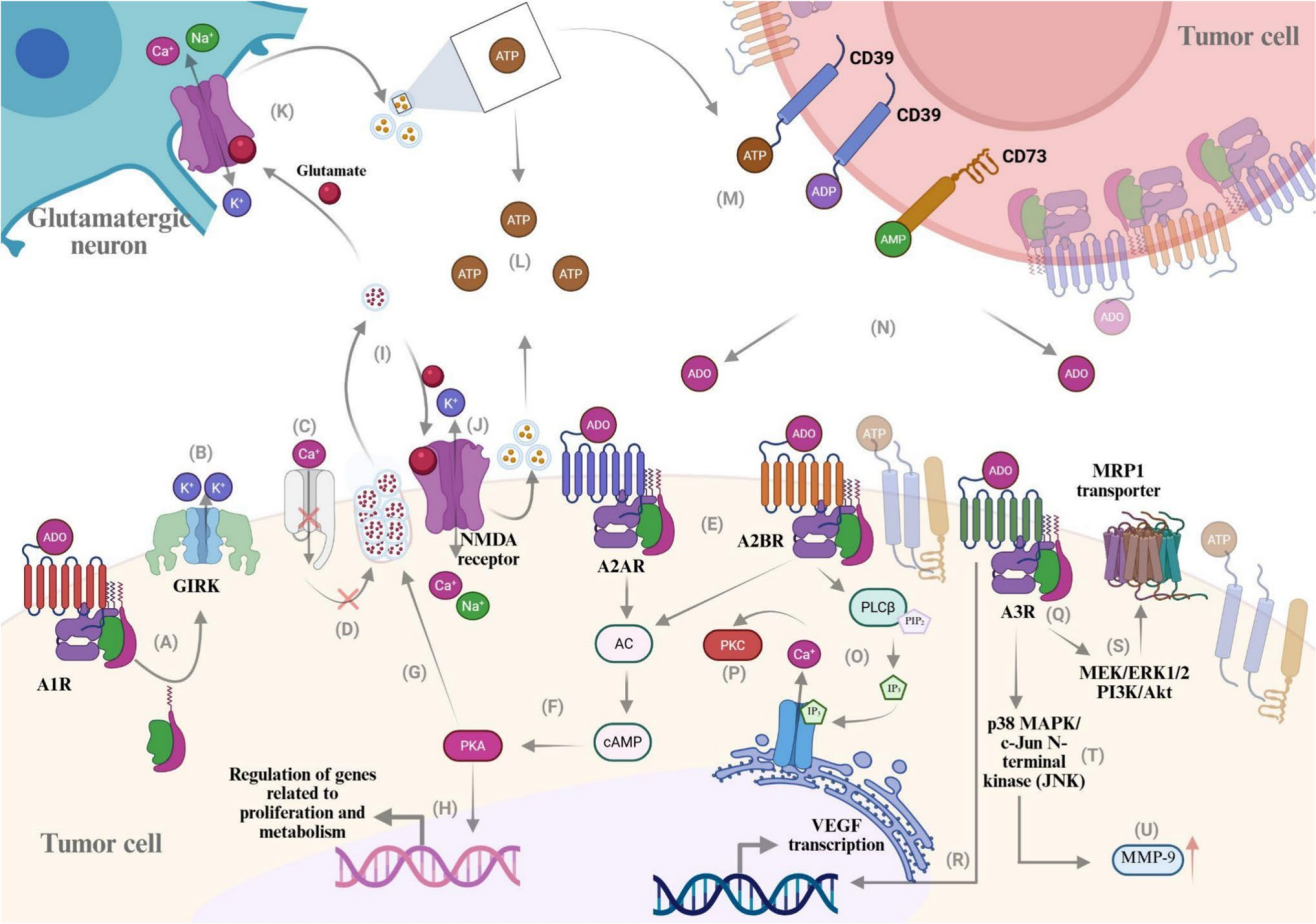
Adenosinergic signaling pathways shaping tumor–neuron interactions and cancer cell behavior in the brain tumor microenvironment. (**A**–**D**) Activation of A1R promotes potassium efflux through GIRK, K_ATP, and SK channels, leading to membrane hyperpolarization and reduced opening of voltage-dependent Ca^2^⁺ channels, ultimately decreasing glutamate release. (**E**–**F**) Activation of A2AR and A2BR stimulates adenylyl cyclase (AC), increasing intracellular cAMP levels and promoting PKA activation. A2BR signaling does not sustain robust PKA activation, as indicated by dashed lines. In contrast, A2AR signaling through the PKA axis enhances vesicular glutamate release, reinforcing excitatory neurotransmission and sustaining NMDA receptor activation in an autocrine/paracrine loop. (**G**–**H**) Reduced glutamatergic signaling limits NMDA receptor activation; however, under sustained stimulation, NMDA receptor activity promotes vesicular release, including ATP-containing vesicles. Extracellular ATP is partially retained in the microenvironment but is predominantly hydrolyzed by ectonucleotidases (CD39/CD73), generating adenosine and contributing to extracellular adenosine (eADO) accumulation. (**I**–**K**, **O**–**P**) A2BR activation engages Gq-dependent signaling via PLCβ, inducing IP₃-mediated Ca^2^⁺ release and PKC activation. These calcium-dependent pathways are associated with cytoskeletal remodeling, invasion, and tumor progression. (**Q**–**U**) A3R activation triggers MAPK/ERK1/2 and PI3K/Akt signaling pathways, contributing to MRP1-mediated drug resistance, and activates p38 MAPK/JNK signaling, leading to increased MMP-9 expression and enhanced migratory capacity. Additionally, A3R signaling promotes VEGF transcription, supporting early neurovascularization. Overall, adenosine receptor (ADOR) signaling integrates neuronal and tumor-derived cues to modulate excitatory signaling, tumor growth, immune evasion, and metastatic potential within the brain tumor microenvironment. Receptors are depicted together for integrative purposes and do not necessarily imply coexpression within the same cell type. Created with BioRender.com

Brain tumors represent some of the most aggressive and clinically challenging neoplasms of the CNS, characterized by profound immune dysregulation and permissive TME. A substantial body of evidence suggests that purinergic signaling, specifically through aADO and its G protein–coupled P1 receptors, plays a pivotal role in the development of these pathological features. Elevated extracellular ADO levels within the TME promote aberrant activation of P1 receptors, leading to suppression of antitumor immune responses, angiogenesis, BBB dysfunction, and resistance to therapy (Fig. 2).

Under pathological conditions, sustained accumulation of extracellular ADO disrupts the physiological balance of P1 receptor engagement, leading to receptor-specific signaling programs that favor tumor progression. Prolonged activation of A2AR and A2BR drives cAMP-dependent pathways that suppress immune effector functions and promote metabolic adaptation, whereas A2BR- and A3R-mediated coupling to calcium- and MAPK-related signaling cascades supports angiogenesis, invasion, and resistance to therapy. In parallel, insufficient A1R-mediated control of excitatory neurotransmission contributes to excitotoxic stress and enhanced ATP release, reinforcing a feed-forward ADO signaling loop within the TME. Collectively, these receptor-dependent mechanisms converge to establish an immunosuppressed, permissive, and treatment-resistant niche that sustains brain tumor progression.

Based on the available evidence, we propose that pathological dysregulation of ADO receptor signaling constitutes a central pro-tumoral mechanism in brain tumors, beyond its association with neuroinflammation, contributing to disease progression and potentially to poor clinical outcomes. A deeper understanding of ADO signaling dynamics in the brain microenvironment, as well as in extracranial tumor sites that precede and support brain metastasis, is therefore essential. In the future, therapeutic strategies should aim to selectively target pathological purinergic signaling. This should be achieved by focusing on receptor-specific modulation and context-dependent interventions. It is also vital to preserve physiological adenosine functions. These are critical for neural homeostasis. This will maximize antitumor efficacy and minimize adverse neurological effects.

## Supplementary Information

Below is the link to the electronic supplementary material.

ESM1 (JPEG 2.09 MB)

ESM2 (JPEG 1.75 MB)

## Data Availability

No datasets were generated or analysed during the current study.
