# Supplementary figures and images for: Adenosine Signaling in Primary and Metastatic Brain Tumors: Immune Suppression, Tumor Progression, and Therapeutic Opportunities
Source: Mol Neurobiol. 2026 May 21;63(1):644. doi: 10.1007/s12035-026-05930-9 (PMC13194290; doi:10.1007/s12035-026-05930-9)

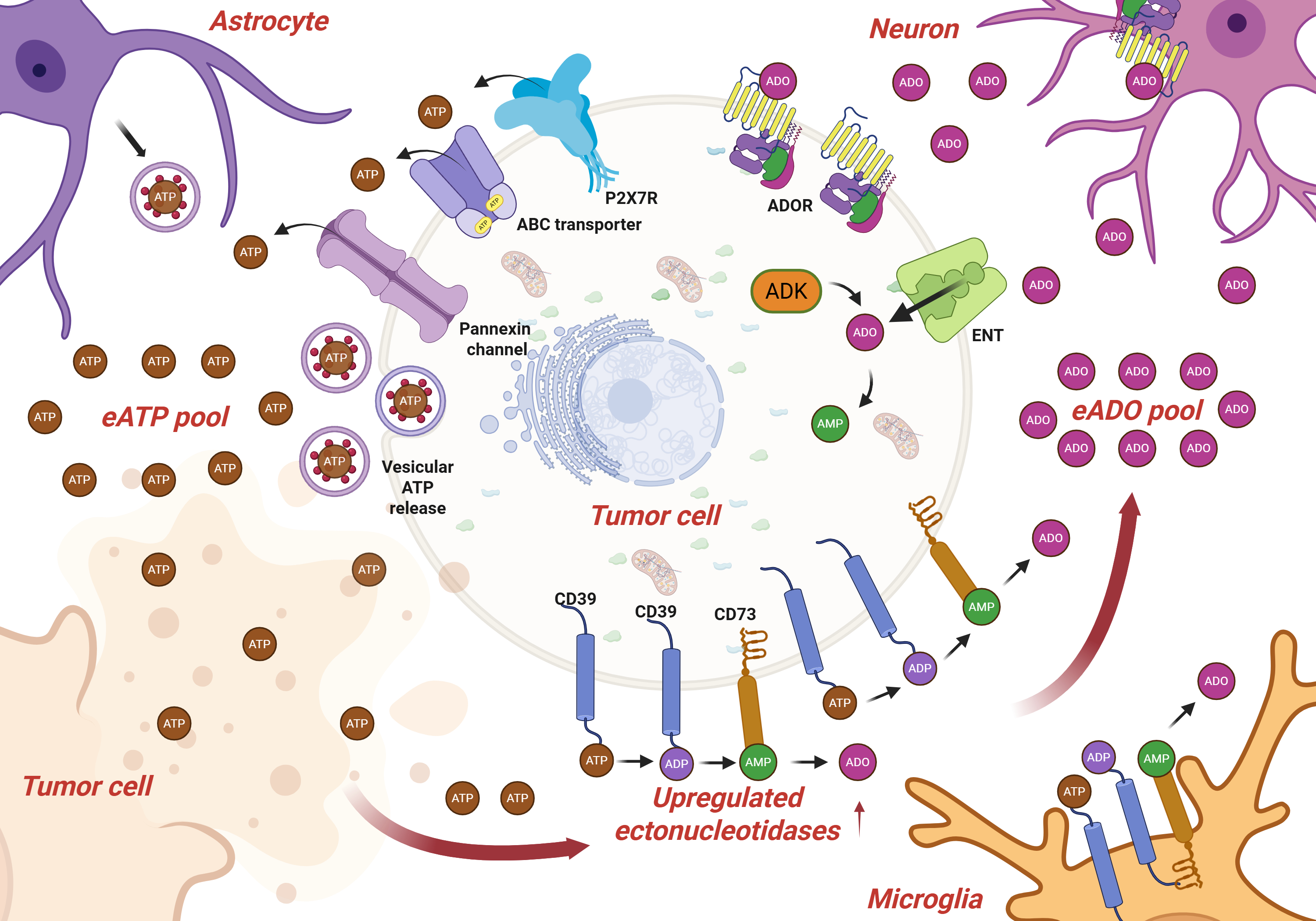

Supplement: Supplementary file 1 — (JPEG 2.09 MB) [file 12035_2026_5930_MOESM1_ESM.jpeg]

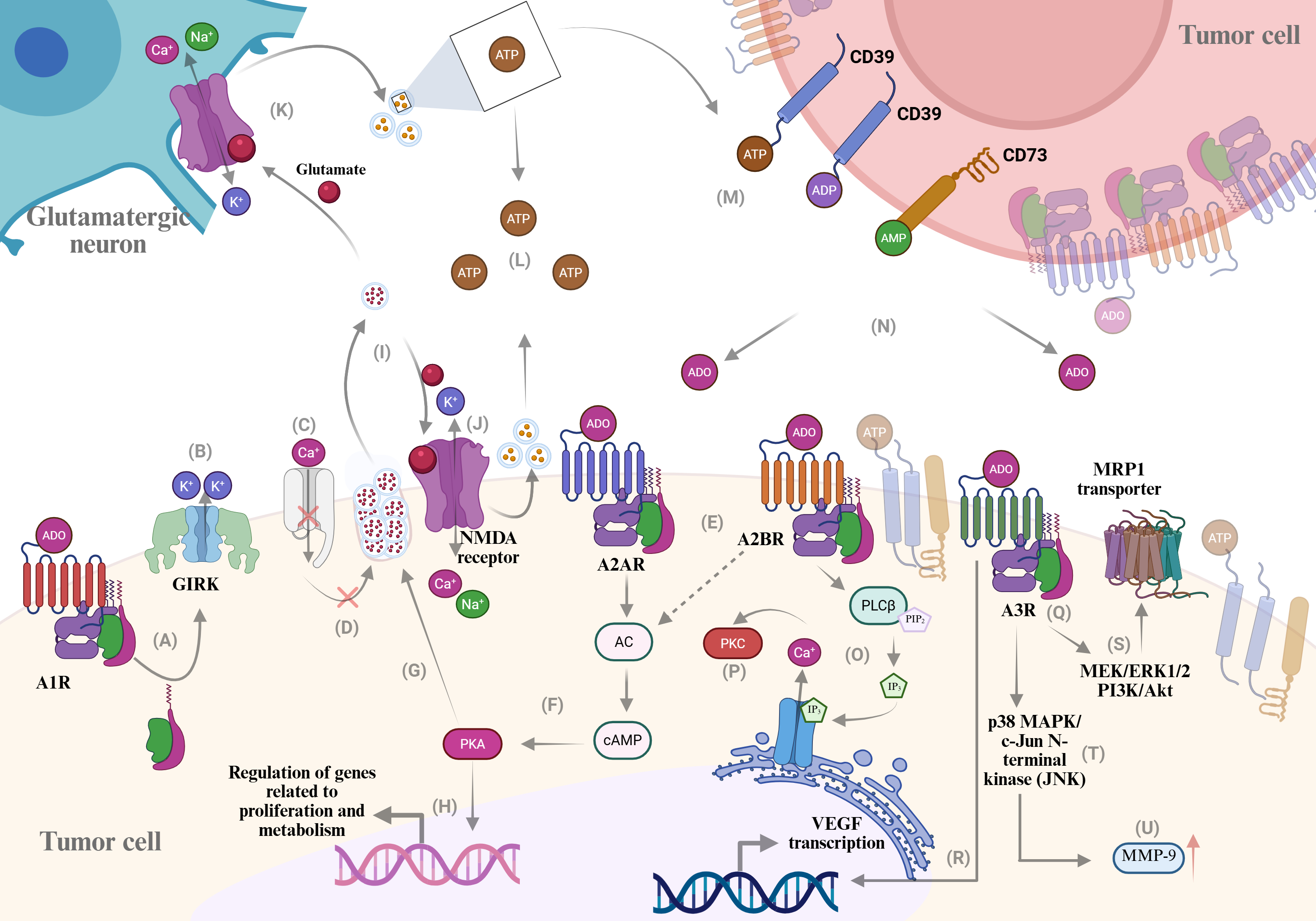

Supplement: Supplementary file 2 — (JPEG 1.75 MB) [file 12035_2026_5930_MOESM2_ESM.jpeg]
